# Bioactivities and Synergistic Effect of *Elsholtzia ciliata* Essential Oil and Its Main Components against *Lasioderma serricorne*

**DOI:** 10.3390/molecules29091924

**Published:** 2024-04-23

**Authors:** Shen Song, Yufei Tang, Rui Feng, Xiaohan Zhang, Yue An, Weibao Kong, Junlong Wang, Ji Zhang, Junyu Liang

**Affiliations:** 1 College of Life Science, Northwest Normal University, Lanzhou 730070, China; 2 New Rural Development Research Institute, Northwest Normal University, Lanzhou 730070, China

**Keywords:** *Elsholtzia ciliata*, *Lasioderma serricorne*, essential oil, carvone, *DL*-limonene, insecticidal activity, synergistic effect, optical isomer, bio-insecticides

## Abstract

Investigations have shown that storage bugs seriously harm grains during storage. In the interim, essential oils (EOs) have been proven to be a good botanical pesticide. The anti-*Lasioderma serricorne* properties of *Elsholtzia ciliata* essential oil, which was obtained by steam distillation, were evaluated using *DL*-limonene, carvone, and their two optical isomer components using contact, repelling, and fumigation techniques. Simultaneously, the fumigation, contact, and repellent activities of carvone and its two optical isomers mixed with *DL*-limonene against *L. serruricorne* were evaluated. The results showed that *E. ciliata,* its main components (*R*-carvone, *DL*-limonene), and *S*-carvone exhibited both fumigations (LC_50_ = 14.47, 4.42, 20.9 and 3.78 mg/L) and contact (LD_50_ = 7.31, 4.03, 28.62 and 5.63 µg/adult) activity against *L.serricorne.* A binary mixture (1:1) of *R*-carvone and *DL*-limonene displayed an obvious synergistic effect. A binary mixture (1:1) of carvone and its two optical isomers exhibited an obvious synergistic effect, too. Furthermore, the repellent activity of the EO, carvone, and its two optical isomers, *DL*-limonene, and a combination of them varied. To stop insect damage during storage, *E. ciliata* and its components can be utilized as bio-insecticides.

## 1. Introduction

*Lasioderma serricorne,* which belongs to Coleoptera, is a destructive storage pest in tropical and subtropical areas of the world, as well as the most widespread warehouse pest [1]. Because of its complicated eating habits, strong fecundity and ability to hide easily in small sizes, it is difficult to prevent and it results in serious economic loss to agriculture [2,3]. Apart from harming many other stored food products and herbs, *L. serricorne* can also infect tobacco, ultimately leading to a decrease in the quality of cigarettes [4]. At present, the commonly used method for preventing warehouse pests is to use chemical pesticide (phosphine) fumigation [5]. Chemical pesticides undoubtedly have a potent poisonous impact, but over time, the misuse of high concentrations of phosphine pesticides can result in major problems, such as drug resistance, pesticide residues, environmental contamination, and other lethal repercussions [6]. For instance, research has linked the risk of adult cancer to the usage of pesticides [7]. Therefore, looking for a new kind of pesticide to replace chemical pesticides is extremely urgent.

Essential oils (EOs) are complex mixtures of volatile organic compounds produced as secondary metabolites in plants [8]. The composition of essential oils is mainly represented by mono- and sesquiterpene hydrocarbons and their oxygenated (hydroxyl and carbonyl) derivatives [9]. Plant isolates contain elements that are poisonous, ovicidal, repellant, and antifeedant to insects. Numerous mechanisms, including touch, ingestion, and the fumigant effect, can cause toxicity [10,11]. Currently, there have been many studies on the contact, fumigation, and repellant activities of essential oils against *L. serricorne*. The literature has reported that *Ajania salicifolia* essential oil presented clear fumigant activity (LC_50_ = 26.60 mg/L) and contact toxicity (LD_50_ = 25.66 μg/adult) against *L. serricorne* [12]. Liang et al. [13] reported that the median lethal dose of *Ajania potaninii* essential oil in *L. serricorne* adults was 22.79 μg/adult; the experimental results of the fumigation activity of *A. potaninii* against *L.serricorne* adults showed that the median lethal concentration was 41.05 mg/L [13]. Also, *Ajania Tenuifolia* essential oil showed potential fumigant toxicity with LC_50_ values of 25.67 mg/L and certain contact toxicity with LD_50_ values of 17.56 μg/adult against *L. serricorne* [14]. Adult *L. serricorne* may be avoided by essential oils from eucalyptus and grapefruit at a concentration of 1 μL/L, with repellent rates of 94.67 and 94.56%, respectively [15].

**Figure 1:**
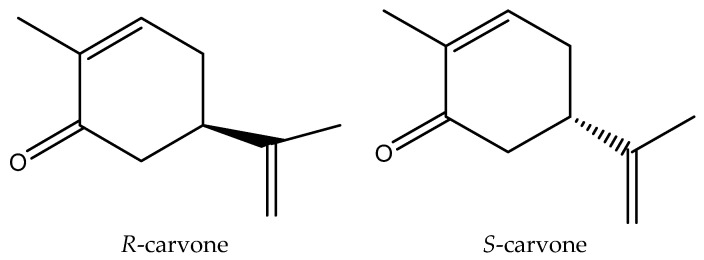
Two structures of carvone.

The genus *Elsholtzia Willd.* of Lamiaceae comprises about 40 species that occur in the tropics, Southern Africa, and subtropics of Asia [16]. EOs of *Elsholtzia fruticosa* are a valuable development of insecticide resources because of the volatile properties and the fact that the smell is easy to diffuse. According to existing research reports, the main components of the aerial parts at the flowering stage of *E. ciliata* essential oil were dehydroelsholtzia ketone (26.5%), carvone (16.6%), elsholtzia ketone (14.6%), and limonene (4.1%) [17]. However, our preliminary research results in the laboratory indicate that carvone and limonene are the two main components of the *E. ciliata* essential oil, with percentages of 31.6% and 22.1%, respectively. The flavoring chemical limonene is frequently found in foods like fruit juices, soft drinks, and pudding [18]. Limonene is a monoterpene hydrocarbon with two optically active forms, *D*- and *L*-limonene. It is a relatively stable terpene that can be racemized to dipentene through heating [19]. Carvone is a monoterpene ketone synthesized by the methylerythritol phosphate pathway in plastids via geranyl-pyrophosphate through an intermediate like limonene or *β*-pinene in some microorganisms and plants [20]. Meanwhile, carvone is also a common component that exists in the EOs of caraway, dill, and spearmint seeds. What is more, it is often used as an additive in pesticides, food, feed, and veterinary medicine [21]. Carvone has two structures, *R*-carvone and *S*-carvone, respectively (Figure 1).

Some studies have shown that *E. ciliata* has an insecticidal effect [17]. For example, the EOs of *Elsholtzia fruticosa* have been proven to have obvious nematicidal activities against *Ditylenchus destructor* (LC_50_ = 0.18, 0.46, 0.66, and 0.38 mg/mL) [22]. It has been found that the *E. ciliata* essential oil has a strong contact toxicity action against *T. castaneum* adults and larvae [10]. Additionally, the fumigant toxicity of *E. ciliate* EOs against *Liposcelis bostrychophila* has been shown. *R*-carvone is the active ingredient in *E. ciliate* essential oils against *Liposcelis bostrychophila*. The LC_50_ of contact toxicity was 57.0 μg/cm^2^, and the fumigant toxicity was 417.4 mg/L, respectively [23]. Thus, it is also necessary to identify the insecticidal activity of compounds in the *E. ciliata* essential oil against *L. serricorne.*

The mixture of two components is often used for research as one of the ways to improve insecticidal activity. Additionally, the combination of pesticides is a part of programs for both integrated pest management (IPM) and insect resistance management (IRM) [24]. Combination pesticide use has been rising in China, making up an estimated 34% of all currently authorized pesticide products [25]. For its use, it is crucial to ascertain the ideal ratio and research the processes of carvone and limonene’s synergy.

This study assesses the toxicity of EO and its primary *E. ciliata* components against *L. serruricorne* for the first time. Simultaneously, research was conducted on the synergistic impact of carvone and its two optical isomers mixed with *DL*-limonene against *L. serricorne*. The results proved the control effects of *E. ciliata* and the main constituents against storage insect pests to provide a basis for the green control of storage pests.

## 2. Results

### 2.1. Chemical Composition Analysis by GC-MS

Preliminary study findings from our lab show that the *E. ciliata* leaf produced around 0.36% (V/m) of essential oil. In total, 16 distinct chemical components were found using the GC-MS method. The three main chemical components identified were limonene (22.1%), *α*-humulene (15.5%), and carvone (31.6%) (Table 1). Among the chemical components of *E. ciliata*, terpenoids are the main components, which make *E. ciliata* have more obvious insecticidal activity. According to published research, terpenoids’ constituents are fast-acting neurotoxins that affect insects [26]. Furthermore, as carvone and limonene are both members of the terpenoids family, these volatiles may contribute significantly to the death rates of pests. Since carvone and *DL*-limonene are *E. ciliata*’s two primary active volatiles, they combine to show notable efficacy against pests. Thus, their bioefficacy against pests (*L. serricorne*) was examined.

### 2.2. Fumigant Toxicity

Table 2 displays the *E. ciliata* EO and its primary ingredients’ efficacy in fumigating *L. serricorne*. The essential oil of *E. ciliata*, *R*-carvone, *S*-carvone, and *DL*-limonene showed obvious fumigation activity against *L. serricorn* with LC_50_ of 14.47 mg/L air, 4.42 mg/L air, 3.78 mg/L air and 20.90 mg/L air, respectively. *R*-carvone’s fumigation activity was approximately 4.73 times greater than *DL*-limonene’s, suggesting that it may have been the main element of the *E. ciliata* EO responsible for the toxicity of the fumigation against *L. serricorne*. Phosphine showed obvious fumigation activity against *L. serricorn* with LC_50_ of 0.08 mg/L air. Although the fumigation activity of the *E. ciliata* EO and its main components against *L. serricorne* was lower than that of positive control phosphine, it had certain advantages compared with other compounds in terms of fumigation activity against *L. serricorne*.

### 2.3. Contact Toxicity

The contact activity of *E. ciliata* EO and its main components on *L. serricorne* is shown in Table 2. The essential oil of *E. ciliata*, *R*-carvone, *S*-carvone and *DL*-limonene showed remarkable contact toxicity against *L. serricorn* with LD_50_ of 7.11 μg/adult, 4.03 μg/adult, 5.63 μg/adult and 28.62 μg/adult, respectively. Furthermore, the contact activity of *R*-carvone was about 7.10 times greater than that of *DL*-limonene and about 1.77 times greater than that of the *E. ciliata* EO, which clearly shows that there is no pattern of one compound being obviously stronger or weaker than the other against the same insect species regarding fumigant toxicity and contact activity. From the data of the contact activity experiment, comparing the negative control to the LD_50_ of pyrethrins is 0.24 μg/adult, the contact activity of *E. ciliata* EO and its main components demonstrated less toxicity against *L. serricorne* adults. However, the essential oil of *E. ciliata* has higher contact toxicity against adult *L. serricorne* compared to other essential oils described in the literature. In addition, the result showed that the contact toxicity of *R*-carvone and *S*-carvone against *L. serricorne* is nearly similiar to LD_50_ of 4.03 μg/adult and 5.63 μg/adult.

### 2.4. Repellency Activity

**Figure 2:**
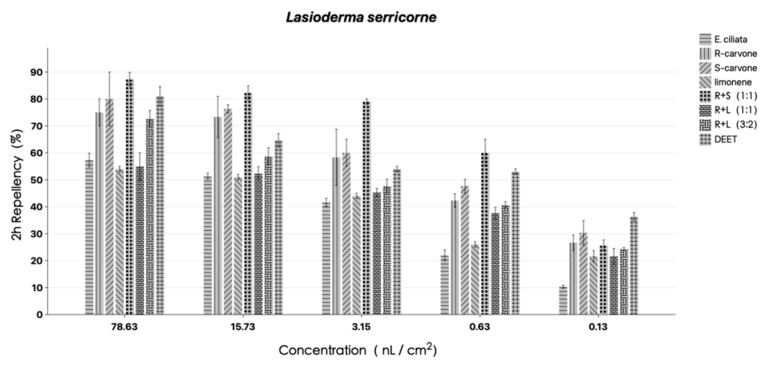
Percentage repellency (PR) of EO, *R*-carvone, *S*-carvone, *DL*-limonene and its mixture isolated from the *E. ciliata* against *L. serricorne* at 2 h after exposure. Differences between PR values of EO and DEET at the same concentration were determined by *t*-test (*p* < 0.05).

**Figure 3:**
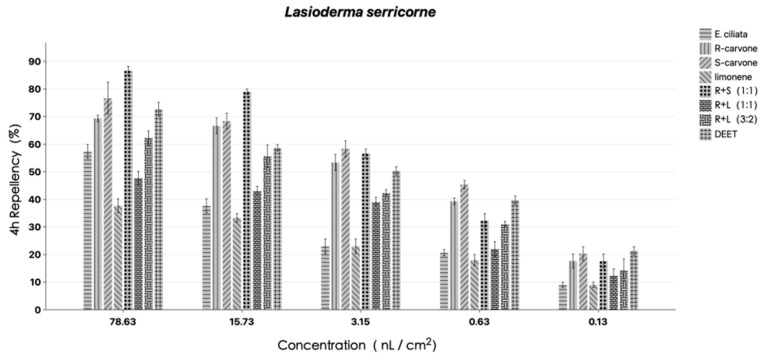
Percentage repellency (PR) of EO, *R*-carvone, *S*-carvone, *DL*-limonene and its mixture isolated from the *E. ciliata* against *L. serricorne* at 4 h after exposure. Differences between PR values of EO and DEET at the same concentration were determined by *t*-test (*p* < 0.05).

Figure 2 and Figure 3 display the results for the repulsive outcomes. The findings demonstrated that varying degrees of repellent action against *L. serricorne* were present in EO, *R*-carvone, *S*-carvone, *DL*-limonene, and their combinations at the testing concentrations. *S*-carvone and its mixture with *R*-carvone (1:1) had PR values ranging from 40–90%, at the same level as DEET, and demonstrated considerable insect repellent action when administered at all tested dosages. At 2 h after exposure, EO, *DL*-limonene, and a combination of *R*-carvone and *DL*-limonene (1:1) all showed only marginal repellent efficacy against *L. serricorne*. Most notably, at the lowest testing concentration, *S*-carvone exhibited strong repellent activity (30 and 20%, respectively) against *L. serricorne* at 2 h after exposure. According to PR values, *R*-carvone and *S*-carvone mixtures (1:1) performed better after 4 h after exposure than EO and other compounds at 78.63 and 15.73 nL/cm^2^, respectively. Regardless of whether exposed for 2 or 4 h, the repellent rate of *S*-carvone is always higher than that of *R*-carvone. But when *R*-carvone is mixed with *DL*-limonene, the repellent rate significantly decreases. It is speculated that *DL*-limonene may have a reducing effect on the efficacy of carvone. Numerous variables, like the sensitivity of the insects, the testing concentration, and the length of exposure, had an easy time influencing the variety of the repellent function. Therefore, more research is required to fully understand the repelling effect.

### 2.5. Synergism of the Two Main Compounds R-Carvone and DL-Limonene against L. serricorne

Table 3 shows the fumigation activity and contact toxicity of *R*-carvone and *DL*-limonene mixed in different ratios against the *L. serricorne*. As the two most abundant compounds in *E.ciliata* essential oil, *R*-carvone was additive with *DL*-limonene when mixed in natural ratios (3:2, R = 1.11) as well as other ratios (1:1, R = 1.36). In particular, an *R*-carvone combination with *DL*-limonene showed a significant increase in its activity over *DL*-limonene. This is consistent with previous results showing that the activity of synergism is somewhere in between the activity of *R*-carvone and *DL*-limonene. Two binary blending ratios’ contact toxicity to *L. serricorne* was assessed. Table 3 also shows how the two mixing ratios worked together. Among the two mixtures, all showed additive effects (the ratio was 1:1 and 3:2). It can be deduced that there were different levels of sensitivity for *L. serricorne* because of the test sample.

### 2.6. Synergetic Effect of Carvone Optical Isomers against L. serricorne

Table 4 shows the fumigation activity of *R*-carvone and *S*-carvone mixed in ratio (1:1) against the *L. serricorne.* Table 4 shows the contact activity of *R*-carvone and *S*-carvone mixed in ratio (1:1) against the *L. serricorne*. Especially, *R*-carvone in a 1:1 ratio (R value of 3.15) combination with *S*-carvone showed a significantly synergistic effect. It is proved that *R*-carvone mixed with *S*-carvone at 1:1 ratio combination was found to have a good effect in terms of fumigant toxicity against *L. serricorne*. As for the contact activity, after mixing *R*-carvone and *S*-carvone in the ratio (1:1), as shown in Table 4, we found that the R value was 0.98, suggesting an addictive effect (0.5 ≤ R ≤ 1.5). Moreover, although the mixture of the two components did not show a synergistic effect, there was no obvious antagonistic interaction among the tested compounds. Therefore, it is expected to develop a new type of botanical pesticides with insecticidal effects on *L. serricorne*.

## 3. Discussion

Different bioactivities of essential oils and their constituents have been used for pharmacological, therapeutic, aromatic, or cosmetic reasons. They are also regarded as a solution for the management of insect pests in stored goods [27]. In this study, the bioactivities and synergistic effect of EO isolated from *E. ciliata* and its main components against *L. serricorne* were researched. The results showed that *E. ciliata* and its main components (*R*-carvone, *DL*-limonene) exhibited repellency, fumigation and contact activity. *S*-carvone, an optical isomer of *R*-carvone, also exhibited similar properties. Furthermore, *R*-carvone and *DL*-limonene exhibited synergistic contact activity at a natural ratio of 3:2 in essential oil. In addition, a binary mixture (1:1) of carvone’s two optical isomers exhibited both fumigant toxicity and contact toxicity.

The terpenoids investigated here were mostly the insecticidal components of EOs. According to literature reports, as typically volatile and fairly lipophilic substances, monoterpenoids can enter insects and affect their physiological processes [28]. They have fumigant qualities due to their high volatility, which may be important for the control of stored-product insects [29]. Despite much study, phosphine fumigation is still necessary to control *L. serricorne* [30]. The actions of their constituent parts determine the insecticidal activity of EOs. Even so, the essential oil’s fumigant efficacy against *L. serricorne* was less potent than that of the widely used fumigant phosphine. However, in comparison to other essential oils used in earlier studies, such as the essential oils of *Ajania salicifolid* and *Ajania potaninii*, which had LC_50_ values of 24.29 mg/L air and 28.67 mg/L air, respectively, and the essential oil of *Agastache foeniculum* (Lamiaceae), which had an LC_50_ value of 21.57 mg/L air, the essential oil of *E. ciliata* showed more significant fumigant toxicity [12,31,32]. It is unknown whether physicochemical or structural characteristics enable compounds to act as repellents. However, recent research has indicated that several characteristics, including lipophilicity, vapor pressure, boiling temperature, molecule length, and the primary moment of inertia, may be useful in describing repellency [33].

Moreover, the insecticidal activity of two optical isomers of carvone was also studied. According to literature reports, the presence of terpenes and a certain degree of lipophilicity might determine toxicity by the interactions with the membrane constituents and their arrangement [28], and there may be some functional group that allows the substance to penetrate the stratum corneum and achieve its targets [34]. Additionally, because carvone is an oxygenated monoterpene, it may contain a toxin that affects pests’ nervous systems [35].

We speculate that the contact and fumigation toxicity of essential oils may be attributed to the synergistic effect of the total activity of pure ingredients. As varied mixing ratios of the separate ingredients may have a synergistic, additive, or antagonistic effect, the ratio may have an impact on the interaction. It is obvious from our experimental data that the toxicity of the mixtures, including *R*-carvone and *DL*-limonene, showed an arresting increase compared with *DL*-limonene, but we found that the LD_50_ values of these mixtures are higher than those of *R*-carvone. Thus, we can presume that *R*-carvone plays an important role in the mixtures, deciding the additive effect of the mixtures including *R*-carvone and *DL*-limonene. At the same time, the activity of the EO is a result of all the components contained in the EO. Thus, it can be inferred that this is why the fumigation and contact activity of *E. ciliata* EO are weaker than those of *R*-carvone. From the perspective of repellency, the mixture of *R*-carvone and *DL*-limonene is lower than that of monomers *R*-carvone or *S*-carvone. One possible reason may be that *DL*-limonene weakens the positive effect of *R*-carvone. On the other hand, the effects of *R*-carvone and *DL*-limonene may be similar, so their combination reduces the contact toxicity of *R*-carvone. Although the combination of the two components did not show a synergistic effect, there was no significant antagonistic interaction between the test substances.

It is worth mentioning that in *E. ciliata* from different regions, the content of the ingredients contained in ciliata varies. For example, the *E. ciliata* essential oil derived from the Mao’er Mountain of Northeastern China mainly stimulated dihydroelsholtzia ketone (68.4%) and elsholtzia token (25.2%) [36]. The use of EO products not from China as a repellent may not result in a positive effect. In addition, *α*-humulene also has insecticidal activity against *L. sericorne*. You et al. isolated *α*-humulene from *Murraya microphylla* branches and leaves, and exhibited the strongest contact activity against *L. sericorne*, showing an LD_50_ value of 13.1 μg/adult [37]. *R*-carvone displayed 2.5 times stronger contact toxicity (no overlap in LC_50_ values) than *E. ciliata* crude essential oil against booklice (*L. bostrychophila*), and dehydroelsholtzia ketone (LC_50_ 151.5 mg/cm^2^) showed the same level of toxicity as the essential oil [20].

From the standpoint of contact toxicity, the EOs from the species reported here are more environmentally friendly. Secondly, their combined use ensures the presence of multiple molecules that can reduce any potential resistance-related issues linked to the use of individual compounds when used as repellents. Overall, the results of this study indicate that *E. ciliata* has a promising future as a bio-insecticide or environmentally friendly pest deterrent for grain and warehouse storage.

## 4. Materials and Methods

### 4.1. Plant Material and EO Isolation

*E. ciliata* was collected from Longxi City (35°10′ N latitude, 104°27′ E longitude, altitude 1880 m) in the Gansu Province of China. The plant species were identified by Dr. Liang, J.Y. (College of Life Science, Northwest Normal University) and then deposited at the Herbarium of College of Life Science, Northwest Normal University. With plant samples of 200 g crushed, the minced sample was connected to the distillation unit, then condensed and maintained for 6 h. Anhydrous sodium sulphate was used to remove excess water after isolation. The resulting dehydrated essential oil was then stored in a dark and airtight glass bottle for refrigeration at 4 °C.

### 4.2. Essential Oil Chemical Characterization Using GC-MS

The gas chromatography–mass spectrometry (GC-MS) technique was employed to unveil the phytochemical constituents of the *E. ciliata* essential oil [10]. The GC-MS analysis was run on an Agilent 6890 N gas chromatograph connected to an Agilent 5973 N mass selective detector (Agilent Technologies, Santa Clara, CA, USA). They were equipped with a gas chromatography–flame ionization detector (GC-FID) and an HP-5MS (30 cm × 0.25 mm × 0.25 µm) capillary column. The essential oil sample was diluted in *n*-hexane to obtain a 1% solution. The injector temperature was maintained at 250 °C with the volume injected being 1 µL. The flow rate of carrier gas (helium) was 1.0 mL/min, with the mass spectra scanned from 50 to 550 *m*/*z*.

Based on RI, the chemical constituents were identified by comparing them with *n*-alkanes as a reference. The components of the essential oil were identified by matching their mass spectra with various computer libraries (Wiley 275 libraries, NIST 05, and RI from other literature, Hoboken, NJ, USA) [38].

### 4.3. Insects

*L. serricorne* were fed in a constant treatment at 30 ± 1 °C, and the relative humidity was 70–80%. The insects were inoculated into a glass container containing wheat flour mixed with yeast (10:1, *w*/*w*). All adults used in the experiment were considered to be at an adult stage after an eclosion time of 1~2 weeks.

### 4.4. Fumigant Toxicity

Liu and Ho’s study looked at the fumigant toxicity of the plant’s *E. ciliata* EO and monomeric chemicals against *L. serricorne* [39]. Ten selected test insects were placed in an empty glass bottle with a diameter of 2.5 cm, 5.5 cm high, and a volume of 25 mL. The EO was diluted with *n*-hexane to obtain five concentration gradients. Diluted liquids of 10 µL were injected into the filter paper (2.0 cm^2^). After 20 s, the bottle was put in an incubator after the cap was secured to create an airtight chamber within. In this study, *n*-hexane was used as a negative control. Moreover, phosphine was used as a positive control. Each concentration was repeated 5 times. The test insects died after 24 h, and the mortality and corrected mortality were estimated after being seen and recorded. The median lethal concentration (LC_50_) was calculated by using probit analysis (SPSS 22.0).

### 4.5. Contact Toxicity

Contact toxicity of the *E. ciliata* essential oil isolated from the plant and monomeric compounds against *L. serricorne* were investigated by Liu and Ho’s [38] method. The EO was diluted with *n*-hexane to obtain five concentration gradients. Insects were paralyzed by using ice packs, and then 0.5 µL diluted liquids were applied to the dorsal thorax of the insects. Ten selected test insects were placed in an empty glass bottle with a diameter of 2.5 cm, 5.5 cm high, and volume of 25 mL. In this study, *n*-Hexane was used as a negative control. Moreover, pyrethrins were used as positive controls. Each concentration was repeated 5 times. After 24 h, the death of the test insects was observed and recorded. The insects were touched with a brush many times; if the insect did not respond, it was dead. The mortality and corrected mortality were calculated. The median lethal concentration (LD_50_) was calculated by using probit analysis (SPSS 22.0).

### 4.6. Repellency Activity

Repellant activity was evaluated using the previously described methodology [40]. The EO, its commercially available compounds (carvone, *DL*-limonene), and a mixture of carvone and *DL*-limonene were dissolved in *n*-hexane to prepare five concentrations (78.63, 15.73, 3.15, 0.63, 0.13 nL/cm^2^). Petri dishes with a 9 cm diameter were utilized as test tubes. Two equal pieces of filter paper with a diameter of 9 cm were cut out. The filter paper was divided in half and covered with 500 µL of a testing solution on one half, and the same amount of *n*-hexane on the other half as a negative control. The filter paper was split in half, flattened, and spread out at the bottom of the Petri dish after spontaneously volatilizing. The Petri dishes were filled with the insects. To ensure that the insects were organically spread, the Petri dishes were then covered with a lid. For each of the five times each treatment was applied, twenty insects were used. N,N-Diethyl-3-methylbenzamide (DEET), a commercial repellent, and *n*-hexane were used as the positive and negative controls, respectively. The *L. serricorne* individuals still present in the treatment group were counted after 2 and 4 h, and the repellence rate was then determined. The value of percent repellency (PR) was calculated using the following formula: PR (%) = [(Nc − Nt)/(Nc + Nt)] × 100.

Nt is the number of insects present in the treated half, whereas Nc is the number of insects present in the negative control half. The average repellent rate was rated by reference at the same time [41], as shown in the table below. With Tukey’s HSD test at *p* < 0.05, PR values were converted into arcsin square root values and submitted to one-way analysis of variance (ANOVA).

### 4.7. Synergistic Experiment

According to Tak et al.s’ evaluation method for mixtures [42], assuming that the half-lethal concentrations of A and B are determined by the virulence of a and b, the potential synergistic interactions between two major compositions carvone and *DL*-limonene could be evaluated. A concentration was selected with 1:1 and the main compositions of essential oils with their natural proportion were 3:2. The contact toxicity and fumigant toxicity methods were selected as before. Five repetitions were carried out for each treatment, and a blank control was set. After 24 h, mortality was recorded; LD_50_ values were estimated as well. By comparing the synergy ratios, the effects of the combinations were classified as synergistic, additive, or antagonistic. Wadley’s statistical model suggests that [35] the expected LD_50_ value of the mixture was calculated from the following equation [43]:

$$\mathrm{Expected}\;{\mathrm{LD}}_{50}=\frac{\mathrm{ana}+b+c+\ldots +n}{\frac{a}{{\mathrm{LD}}_{50\left(a\right)}}+\frac{b}{{\mathrm{LD}}_{50\left(b\right)}}+\frac{c}{{\mathrm{LD}}_{50\left(c\right)}}+\ldots +\frac{n}{{\mathrm{LD}}_{50\left(n\right)}}}$$

where a represents the amount of compound A in the mixture and LD_50_ (a) represents the actual LD_50_ value for compound A. The following was determined for the values of R, which represent the relationships between the chemicals in the mixture:

$$R=\frac{{\mathrm{expected}\;\mathrm{LD}}_{50}}{{\mathrm{observed}\;\mathrm{LD}}_{50}}$$

Values of R > 1.5 indicated the synergistic action between the compounds of the mixture against *L. serricorne*, whereas values of 1.5 ≥ R > 0.5 or R ≤ 0.5 were considered to show additive or antagonistic interaction against *L. Serricorne,* respectively.

### 4.8. Chemicals

Pyrethrins were purchased from Dr. Ehrenstorfer GmbH, Augsburg, Germany with a concentration of 27%. Phoxim was purchased from Dr. Ehrenstorfer GmbH, Augsburg, Germany with a purity of 98.0%. *R*-carvone were purchased from Tokyo Chemical Industry Co., Ltd., Tokyo, Japan, with a purity of 99%. *S*-carvone was purchased from Shanghai Aladdin Biochemical Technology Co., Ltd., Shanghai, China, with a concentration of 97%. *DL*-limonene was purchased from Beijing Yinokai Technology Co., Ltd., Beijing, China, with a purity of 95.0%.

## 5. Conclusions

In comparison to pyrethrins and phosphine, the EOs from *E. ciliata,* as well as its primary constituents, have a greater ability to operate as fumigants and contact toxins against *L. serricorne*. *R*-carvone and *DL*-limonene together in a 3:2 ratio provide an effective repellant. It is strongly recommended to combine these EOs in formulations of bio-insecticides that are favorable to the environment.

## Figures and Tables

**Table 1:** The major chemical constituents of *E. ciliata* leaf oil *.

Number	Constituent	Retention Time/Min (Rt)	Ri **	Relative Content (%)
1	Limonene	4.464	1040	22.1
2	*β*-ocimene	4.654	1061	4.1
3	Carvone	7.366	1216	31.6
4	Dehydroelsholtzia ketone	8.104	1277	14.9
5	(E)-*β*-caryophllene	10.077	1414	2.9
6	*α*-humulene	10.397	1450	15.5

* date from reference [10]. ** RI (retention index) as determined on an HP-5MS column using the homologous series of n-hydrocarbons.

**Table 2:** Fumigant toxicity and contact toxicity of *E. ciliata* EO and its main constituents against *L. serricorne* adults.

Experiment	Treatment	LC50 (mg/L Air)	95% FL (mg/L Air)	Slope ± SE	Chi Square	*p* Value
Fumigant toxicity	*E. ciliata* EO	14.47	13.14~15.87	8.33 ± 1.40	8.44	0.98
	*R*-carvone	4.42	3.41~5.14	6.11 ± 1.52	2.9	0.89
	*S*-carvone	3.78	3.38~4.61	5.02 ± 1.59	2.44	0.93
	*DL*-limonene	20.9	15.79~26.43	0.21 ± 0.05	1.92	0.96
	Phosphine *	0.08	0.07~0.11	4.06 ± 0.31	6.27	0.97
Contact toxicity	**Treatment**	**LD50 (μg/Adult)**	**95% FL (μg/Adult)**	**Slope ± SE**	**Chi Square**	***p* Value**
	*E. ciliata* EO	7.13	6.30~8.12	1.29 ± 0.18	6.68	0.92
	*R*-carvone	4.03	3.20~4.81	2.17 ± 0.54	0.73	0.99
	*S*-carvone	5.63	4.83~6.72	1.91 ± 0.39	1.75	1.00
	*DL*-limonene	28.62	23.23~35.05	0.24 ± 0.05	2.03	0.96
	pyrethrins	0.24	0.16~0.35	1.31 ± 0.20	17.36	0.8

Phosphine * date from reference [13].

**Table 3:** Fumigation toxicity and contact toxicity of *R*-carvone and *DL*-limonene mixture against *L. serricorne*.

Experiment	Volume Ratio	Observed LC_50_ (mg/L air)	Slope ± SE	*p* Value	Expected LC_50_ (μg/Adult)	R ^a^	Note
Fumigant toxicity	1:1	5.38	1.39 ± 0.33	1	7.32	1.36	Additive
	3:2	5.82	1.91 ± 0.34	0.53	6.46	1.11	Additive
Contact toxicity	**Volume Ratio**	**Observed LD_50_** **(μg/Adult)**	**Slope ± SE**	***p* Value**	**Expected LD_50_** **(μg/Adult)**	**R ^a^**	**Note**
	1:1	14.4	0.53 ± 0.13	0.93	7.33	0.51	Additive
	3:2	9.547	0.70 ± 0.15	0.66	6.14	0.64	Additive

^a^ The effect of mixtures was defined as synergistic when R > 1.5; additive when 0.5 ≤ R ≤ 1.5; antagonistic when R < 0.5.

**Table 4:** Fumigation toxicity and contact toxicity of *R*-carvone and *S*-carvone mixture against *L. serricorne*.

Experiment	Volume Ratio	Observed LC50 (mg/L Air)	Slope ± SE	*p* Value	Expected LC50 (μg/Adult)	R ^a^	Note
Fumigant toxicity	1:1	1.30	3.60 ± 0.99	1	4.08	3.15	Synergistic
Contact toxicity	**Volume Ratio**	**Observed LD50** **(μg/Adult)**	**Slope ± SE**	***p* Value**	**Expected LD50** **(μg/Adult)**	**R ^a^**	**Note**
	1:1	4.80	1.99 ± 0.49	0.59	4.70	0.98	Synergistic

^a^ The effect of mixtures was defined as synergistic when R > 1.5; additive when 0.5 ≤ R ≤ 1.5; antagonistic when R < 0.5.

## Data Availability

Data are contained within the article.
